# The epidemiology of medically attended respiratory syncytial virus in older adults in the United States: A systematic review

**DOI:** 10.1371/journal.pone.0182321

**Published:** 2017-08-10

**Authors:** Ann D. Colosia, Jin Yang, Eric Hillson, Josephine Mauskopf, Catherine Copley-Merriman, Vivek Shinde, Jeffrey Stoddard

**Affiliations:** 1 RTI Health Solutions, Research Triangle Park, North Carolina, United States; 2 Novavax, Gaithersburg, Maryland, United States; University of Calgary, CANADA

## Abstract

**Objective:**

This review was undertaken to assess the historical evidence of the disease incidence and burden of laboratory-confirmed respiratory syncytial virus (RSV) in medically attended older adults.

**Design:**

A qualitative systematic literature review was performed; no statistical synthesis of the data was planned, in anticipation of expected heterogeneity across studies in this population.

**Methods:**

A literature search of PubMed, Embase, and the Cochrane Library was conducted for studies of medically attended RSV in older adults (≥ 50 years) published in the last 15 years. Two independent reviewers screened titles and abstracts based on predefined inclusion and exclusion criteria.

**Results:**

From 10 studies reporting incidence proportions, RSV may be the causative agent in up to 12% of medically attended acute respiratory illness in older adults unselected for comorbidities, with variations in clinical setting and by year. In multiple studies, medically attended–RSV incidence among older adults not selected for having underlying health conditions increased with increasing age. Of prospectively followed lung transplant recipients, 16% tested positive for RSV. In hospitalized adults with chronic cardiopulmonary diseases, 8% to 13% were infected with RSV during winter seasons (8%-13%) or metapneumovirus season (8%). Hospitalizations for RSV in older adults typically lasted 3 to 6 days, with substantial proportions requiring intensive care unit admission and mechanical ventilation. Among older adults hospitalized with RSV, the mortality rate was 6% to 8%.

**Conclusions:**

Protection of older adults against RSV could reduce respiratory-related burden, especially as age increases and the prevalence of comorbidities (especially cardiopulmonary comorbidities) grows.

## Introduction

Respiratory syncytial virus (RSV) has long been known to be a major pathogen in young children [1, 2], but there is growing recognition of the threat of RSV in older adults [3]. RSV outbreaks have been documented in long-term care facilities [4, 5] and senior daycare programs [6]. Older adults living in the general community are also susceptible to respiratory illness from RSV, with subsequent increased risk of emergency department (ED) visits [7] or hospitalization [7–9]. Growing recognition of the need to protect older adults from RSV complications has led to efforts to develop a vaccine for this population [10–13]. However, development of an RSV vaccine for any age group has been in progress since the 1960s and has proven difficult. A successful vaccine would need to stimulate a robust, long-lasting immune response that is superior to the partial, transient immunity conferred by natural infection [14]. There is currently no approved RSV vaccine for older adults or any other population in the United States (US) or globally.

The symptoms of RSV are not readily distinguished from other respiratory viral infections. Although nasal congestion (indicative of an upper respiratory tract infection), wheezing (indicative of a lower respiratory tract infection), and fever (more common in influenza infections than RSV infections[3]) are independent predictors of RSV in older adults, having all three symptoms has poor sensitivity for diagnosing RSV [15].

Antiviral therapy approved for treatment of RSV in older adults is also currently not available, and treatments are symptomatic and/or supportive. Symptomatic medicines for RSV include bronchodilators and inhaled corticosteroids, especially for individuals with asthma or chronic obstructive pulmonary disease (COPD). If hospitalized, supportive care may include fluids, supplemental oxygen [16], or mechanical ventilation for serious disease [3].

Estimating the true incidence of RSV in older adults seeking medical care for a respiratory illness is associated with challenging logistical issues (e.g., delayed presentation for clinical illness rendering virological detection and confirmation of diagnosis more challenging [17], and need for repeat testing in the acute and convalescent phases for serologic analysis), testing difficulties (e.g., need for special equipment for polymerase chain reaction [PCR] of viral panel assays [18] and the increased cost associated with routine PCR testing)[19], and low health care provider awareness of RSV as an important cause of viral respiratory disease in older adults. Prior to the wide availability of PCR assays, non-PCR methods had low and/or variable sensitivity in adults [3, 18]. In addition, with limited treatment options, there has been little incentive for physicians to test for RSV. Therefore, prospective studies designed to assess viral etiology are the most robust source of RSV incidence in older adults.

Another difficulty in determining RSV incidence in older adults is that RSV transmission occurs seasonally, primarily in the winter in the US, and the incidence can vary from year to year [20] and geographically [21].

This systematic literature review was undertaken to assess the recent evidence on the burden of laboratory-confirmed RSV in medically attended older adults. The results reported here focus on adults aged 50 or more years with medically attended RSV in the US and studies that reported on the incidence, prevalence, mortality, and/or resource use in the US.

## Methods

Based on a predefined protocol (available upon request), a literature search of PubMed (see S1 Table in the Supporting Information), Embase, and Cochrane was conducted for articles summarizing studies of the burden of laboratory-confirmed RSV in medically attended older adults. A search strategy was constructed using a combination of Medical Subject Headings or Emtree terms and free-text terms in titles or as text words (S1 Table in Supporting Information shows the PubMed search strategy). The search and inclusion criteria had no country or language restriction. Search dates for recent primary studies were January 1, 2000, to March 11, 2016. Initially, relevant review articles were retained for bibliography review if published from January 1, 2010, to March 11, 2016. Bibliographies of included primary studies and key review articles were examined for studies published from 2000 to 2016 that were not captured in the database search. Conference abstracts from 2014 to 2015 were retrieved from Embase for the American Thoracic Society, the European Respiratory Society, and the International Society for Pharmacoeconomics and Outcomes Research.

Retrieved titles and abstracts (level1) and then selected full-text articles (level 2) were screened independently by two reviewers. A senior researcher served as arbiter when consensus was not reached. Full-text articles not published in English were also screened by a third screener fluent in that language. Included studies were prospective or retrospective, observational, cohort, case-control, or randomized studies or systematic literature reviews with meta-analyses published from 2000 to 2016; case studies and case series were not included. There was no restriction based on sample size. However, for mortality from RSV, data were presented only if at least 10 patients had RSV. Although older adults might be better defined as aged ≥ 60 years, many studies used 50 to 64 as one of the age subsets. Therefore, included studies assessed RSV burden in patients who were aged ≥ 50 years, or if the mean or median age was ≥ 50 years. Medically attended RSV was defined as laboratory confirmation (any method) of RSV during a health care encounter (office visit, ED visit, or hospitalization). A further screen was performed to identify those studies that reported data for the US population.

Data extracted from the US studies by one researcher were verified independently by a second researcher. Extracted data included sampling and testing method for diagnosing RSV, clinical setting, study design, study period, defined patient population, and outcomes of interest. The following outcomes were extracted: incidence per 10,000 persons in the population of interest; incidence proportion, defined as the percentage of those tested for RSV who tested positive; and resource use and mortality rates for adults with RSV. Incidence data were extracted for total RSV (RSV-A plus RSV-B) when available, and for RSV-A and RSV-B separately when combined data were not provided. Populations of interest were the general population, populations with acute respiratory infection symptoms, and populations with comorbid conditions.

Questions relevant to this review from critical appraisal checklists issued by the Scottish Intercollegiate Guidelines Network [22] were answered to assess the quality of the included studies (see S2 through S4 Tables in the Supporting Information).

## Results and discussion

### Literature review results

**Fig 1:**
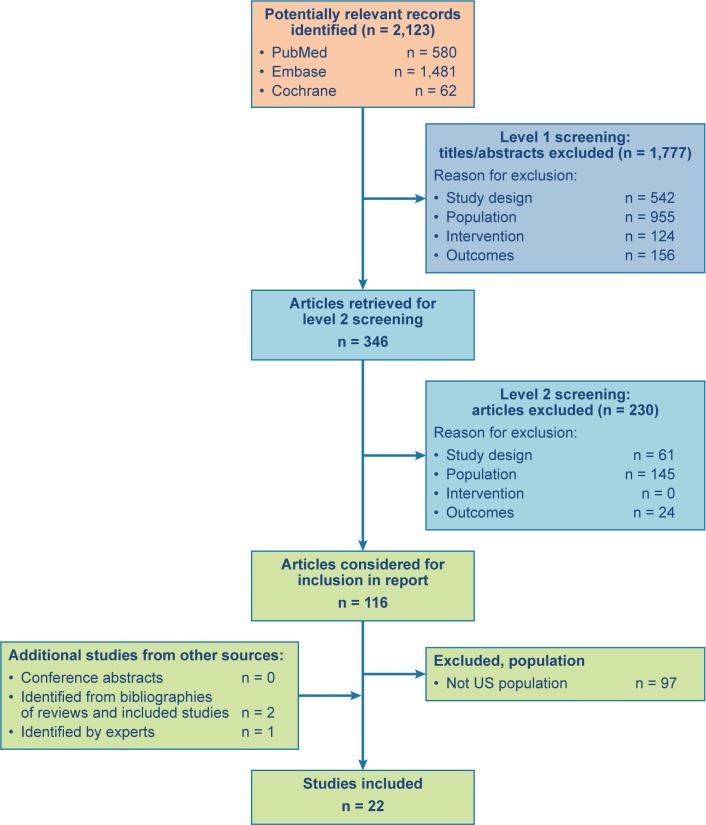
PRISMA diagram. PRISMA = Preferred Reporting Items for Systematic Reviews and Meta-Analyses.

A literature search of PubMed, Embase, and Cochrane identified 2,123 unique articles. Of these, 1,777 articles were excluded during screening title/abstract review, and 346 articles were included for full-text review. Of the 346 articles evaluated during level 2 screening, 230 were excluded, and 116 met the inclusion criteria (Fig 1). For this paper, a further 97 studies were excluded because they did not provide data for the US, leaving 19 studies with US data [7, 12, 17, 20, 23–37]. Additional screening of conference abstracts and bibliographies and consultation with disease experts added three studies with US data [38–40], for a total of 22 included studies.

### Critical appraisal overview

In Supporting Information, S2 through S4 Tables show the results of the critical appraisal of the individual studies by outcome. By design, all of the studies included in this review used laboratory methods to test for RSV rather than statistical modeling of RSV-attributable diagnoses of respiratory illness. PCR and/or serology were the most sensitive [18] and most common methods to detect RSV. Incidence and prevalence were generally reported for older age subgroups within unselected patient populations, whereas the studies in special populations were included based on high mean or median ages among adults of all ages. Confidence intervals (CIs) were often not reported. Most of the studies were prospective; only three were retrospective studies [26, 32, 33], and one of those was a retrospective analysis of prospectively followed patients [33]. Two-thirds of the studies were multicenter [7, 12, 17, 20, 23, 24, 26, 27, 29–32, 38, 40], and only half were large (> 300 persons) regarding the older adult populations assessed for RSV outcomes [7, 12, 17, 20, 23, 24, 27, 29, 30, 39, 40]. Only one study [17] claimed to have a nationally representative patient population.

### Incidence of medically attended RSV in unselected populations by season and clinical setting

The incidence of RSV in unselected populations of older adults was reported for varying clinical settings outside and within the hospital. These older adults included both those with and without underlying chronic conditions. The studies differed in their presentation of incidence: some reported *incidence rates* (number of new cases per population at risk within a specified period) [17, 20, 23, 24, 40], and some reported *incidence proportions* (number of new cases of RSV infection among a population at risk without the disease at baseline expressed as a percentage of acute respiratory illness [ARI] cases observed in the population) [23–29, 31, 39, 40].

#### Incidence rate of medically attended RSV

**Fig 2:**
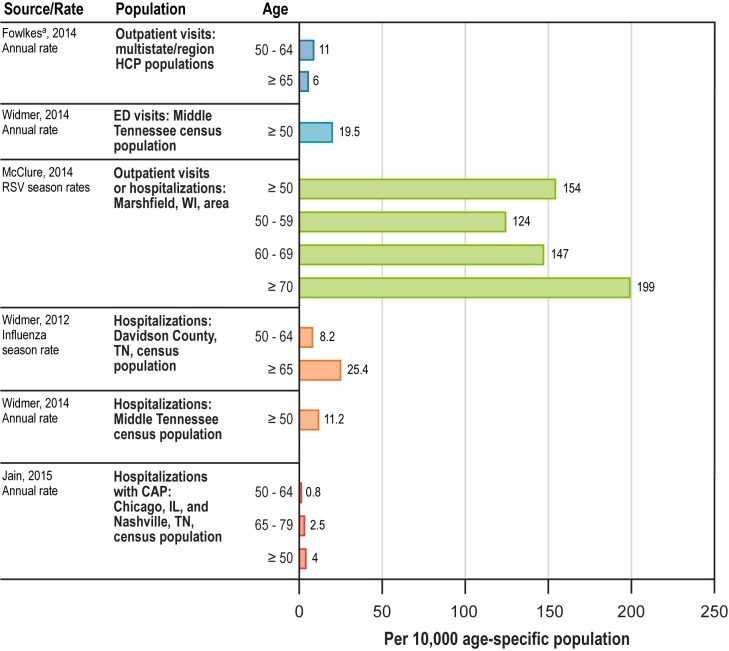
Incidence rates for medically attended RSV for age-specific populations by type of RSV medical encounter. CAP = community-acquired pneumonia; ED = emergency department; HCP = health care provider; RSV = respiratory syncytial virus. ^a^ In Fowlkes et al. [17], surveillance was conducted in 57 HCP practices in 12 sites, including nine states (Florida, Iowa, Minnesota, New Jersey, North Dakota, Oregon, Utah, Virginia, and Wisconsin) and three jurisdictions (Los Angeles County, New York City, and Philadelphia).

Five prospective US studies reported incidence rates of medically attended RSV in the overall population: one in an outpatient setting by Fowlkes et al. [17], one in the outpatient or inpatient setting by McClure et al. [20], one in the ED or inpatient setting by Widmer et al. [23], and two in the inpatient setting by Jain et al. [40] and Widmer et al. [24] (Fig 2). McClure et al. [20] presented seasonal rates; the other studies stated that they reported annual rates.

Despite differences in methodology and medically attended-RSV incidence rates in the studies, the outcomes were generally consistent in showing higher incidence rates with increasing age. An exception was the outpatient study by Fowlkes et al. [17]. The outpatient estimate in this study may be affected by willingness to attend, or access to, outpatient care across the older age subgroups, including the traditional health care practices that made up most of the study sites in Fowlkes et al. [17]. Fig 2 shows the medically attended–RSV incidence rates by age category expressed as per 10,000 persons. The clinical presentation in older adults differs from the classic symptoms of RSV found in children, and compared to children, older adults have lower viral titers, which inhibits detection by rapid antigen tests [18], leading to underdiagnosis of RSV in the elderly by surveillance using this method [17]. The annual incidence of medically attended–RSV infection in Fowlkes’ 12 nationally representative US sites was 11.0 per 10,000 for ages 50–64 years and 6.0 per 10,000 for ages ≥ 65 years [17]. Fowlkes extrapolated from those tested to all patients registered at the clinical sites. The McClure et al. [20] study in a single site in Wisconsin, which included both outpatients and inpatients in the influenza season, assumed the same proportion of older adults in the community had RSV as in the tested population, and extrapolated again to account for RSV rates in older adults in the part of the RSV season excluded from their study to the entire RSV season using RSV case rates that occurred mostly in children. After these extrapolations, McClure et al. [20] showed a much higher rate of outpatient visits and an increase with age (ages 50–59 years, 124 per 10,000 per season; ages 60–69 years, 147 per 10,000 per season; ages ≥ 70 years, 199 per 10,000 per season) [20]. In a study in middle Tennessee by Widmer et al. [23], extrapolating from the proportion of county respiratory infections at five surveillance sites, the RSV hospitalization rate for Davidson county and surrounding counties was 11.24 per 10,000 community residents aged ≥ 50 years [23]. Another middle Tennessee study, using a similar extrapolation method but in different seasons from the first study, showed an increase of RSV disease with increasing age, with annual estimates of 8.2 per 10,000 persons aged 50–64 years and 25.4 per 10,000 persons aged ≥ 65 years [24]. Finally, in a study by Jain et al. [40] of older adults hospitalized with RSV community-acquired pneumonia (CAP), extrapolating from surveillance in five hospitals to the estimated population for the catchment areas, the annual incidence was 0.8 per 10,000 for ages 50–64 years, 2.5 per 10,000 for ages 65–79 years, and 4.0 per 10,000 for ages ≥ 80 years [40].

All of the studies reporting incidence rates of medically attended RSV were large, prospective, multicenter studies that used sensitive RSV detection methods. However, the case definitions that resulted in testing for RSV differed. Most obviously, the studies differed by the clinical setting in which the patients were seen and the observation periods (Fig 2). For example, the population in Fowlkes et al. [17] primarily consisted of patients seen by primary care providers, and only 2 of the 57 participating facilities were emergency or urgent care facilities, limiting the types of health care sites for detecting RSV. The other studies were limited in points of care to the hospital [24, 40] or to the ED and hospital, with rates reported separately in Widmer et al. [23]. Year-long observational periods potentially will include higher proportions of older adults with ARI due to non-RSV infection because RSV is usually confined to several months in the winter. This would be expected to decrease the proportion of RSV-positive cases and lower the estimated incidence rate of RSV infection per person-year compared with the incidence rate per person-seasons from a study limited to the typical RSV season or to an influenza season. RSV incidence determined during the influenza period may omit some of the months when RSV is circulating because the two viral seasons do not completely overlap, also causing RSV incidence to appear lower. Only two of the studies were in the same clinical setting (hospitalized [and CAP not required]) [23, 24], and these studies were conducted in the same geographic region by the same investigators but in different years; their results are roughly consistent given the different age breakouts and potentially different rates in different time periods (Fig 2).

The studies also differed in their definitions of ARI. In the only study that exclusively included outpatients, Fowlkes et al. [17] enrolled patients who had influenza-like illness, defined as fever with cough or sore throat, or ARI, defined as two or more respiratory symptoms, including fever, cough, sore throat, nasal congestion, or rhinorrhea, not meeting the definition of influenza-like illness. Many of the cases of RSV in older adults did not meet the influenza-like illness definition, which required fever to be present, whereas fever is absent in a substantial proportion of older adults with RSV infection [8, 15, 24, 29, 41, 42]. The rate of medically attended RSV in adults with influenza-like illness aged 50 to 64 years was 3 per 10,000 persons, and no cases were detected among adults aged ≥ 65 years with influenza-like illness. The medically attended RSV incidence rate among ARI cases was substantially more sensitive to RSV detection and higher at 5 per 10,000 adults aged 50 to 64 years and 7 per 10,000 adults aged ≥ 65 years [17]. In the outpatient and inpatient study by McClure et al. [20], patients were enrolled who had acute respiratory symptoms with fever/feverishness, chills, or cough and so might have had fewer symptoms than the two required in the Fowlkes study [17]. In other studies reporting medically attended RSV rates, fever was among the symptoms defining acute respiratory illness but was not required for inclusion. The all-hospitalized study by Widmer et al. [24] enrolled patients who had respiratory symptoms or nonlocalizing fever. The other all-hospitalized study, by Jain et al. [40], enrolled patients with ARI and chest-x-ray–confirmed CAP [40]. In the study with inpatients and ED visits by Widmer et al. [23], patients had respiratory symptoms or nonlocalizing fever [23].

Only the Fowlkes et al. [17] outpatient study included geographically diverse patients, with study centers spread across the US in nine states (Florida, Iowa, Minnesota, New Jersey, North Dakota, Oregon, Utah, Virginia, and Wisconsin) and three metropolitan areas (Los Angeles County, New York City, and Philadelphia) [17]. The Jain et al. [40] study of patients hospitalized with CAP was conducted in two locations in the northern US and southeastern US (Chicago, Illinois, and Nashville, Tennessee) [40]. The other studies reporting RSV incidence rates were limited to single locations in the northern US (Marshfield, Wisconsin, in McClure et al. [20] or the southeastern US [Middle Tennessee] in Widmer et al. [23] and Widmer et al. [24]).

#### Incidence proportion of medically attended RSV

Ten US studies reported medically attended–RSV incidence proportions in populations with various manifestations of ARI as outpatients or inpatients (Table 1) [23–29, 31, 39, 40]. The overall conclusion from reviewing these studies is that RSV may be the causative agent in a substantial proportion of medically attended ARI in older adults, but the proportion affected is likely to be less than 15%. However, the proportion of older adults with ARI who tested positive for RSV infection appears to be higher as age increases. For example, Walker and Ison [26] reported that the proportion of RSV-positive patients increased with each 10-year age group, from 7.3% of ages 50–59 years, 12.2% of ages 60–69 years, 14.3% of ages 70–79 years, and 38.7% of ages 80–89 years [26] (Table 1).

**Table 1:** RSV incidence proportions by observational period and clinical setting in unselected populations of older adults.

Reference	RSV Test	Study Location, Design	Dates	Population	Age in Years (Range, Median [M], or Average [A])	N	Incidence (Percentage of Patients)		
							0%-5%	> 5%-10%	> 10%
**RSV Testing Throughout Year**									
**Mix of Clinical Settings**									
Widmer et al. [23]	PCR	Middle Tennessee; Prospective cohort; Multicenter	2009–2010 (12 months)	Respiratory symptoms or nonlocalizing fever seen at ED or hospital	≥ 50; M, 60.8	644	3.1%		
**Hospitalized**									
Sumino et al. [25]	PCR, 100%; DFA, 70%	St. Louis, MO; Prospective^a^ cohort	Oct 2005-Oct 2006	Patients hospitalized with ARI and had bronchoscopy and microbiological testing of BAL	A, 55	283	2.1%		
Walker and Ison [26]	PCR	Chicago, IL; Retrospective cohort; Multicenter	Apr 1, 2009-Mar 31, 2010	Tested positive on molecular respiratory viral panel assay or assay for influenza and RSV	≥ 50	264			14.4%
					50–59	96		7.3%	
					60–69	90			12.2%
					70–79	42			14.3%
					80–89	31			38.7%
					≥ 90	5			40.0%
Glezen et al. [31]	Serology/‌viral culture	Harris County, TX; Prospective; Multicenter	July 1991-June 1995	Patients hospitalized with acute respiratory conditions	≥ 65	150	1.3%		
Jain et al. [40]	PCR, 99.5%; Serology, 37.8%	Chicago, IL, and Nashville, TN; Prospective cohort; Multicenter	July 2010-June 2012	CRX-confirmed CAP requiring hospitalization (excluded severely immunocompromised)	50–64	773	3%		
					65–79	506	4%		
					≥ 80	299	4%		
**ICU**									
Wiemken et al. [27]	PCR	Kentucky, 6 diverse geographic regions; Prospective cohort; Multicenter	Dec 2008-Oct 2011	Patients admitted to ICU with CAP	Average, 52	468	RSV A, 4%; RSV B, 4%		
**RSV Testing During Influenza Season**									
**Nonnational, Outpatients (not ED)**									
Zimmerman et al. [28]	PCR	Pittsburgh, PA; Prospective cohort; Single center	Jan-Apr 2012; (influenza season)	Outpatients only with upper respiratory tract illness	≥ 50	142		8.5%	
**Mix of Clinical Settings**									
Sundaram et al. [29]	PCR	Marshfield, WI, region; Prospective cohort; Multicenter	2004–2010 influenza seasons	Outpatients or inpatients seen for ARI	≥ 50; A, 64.3	2,225		9.2%	
**Hospitalized**									
Widmer et al. [24]	PCR	Davidson County (Nashville), TN; Prospective cohort; Multicenter	2006–2009 influenza seasons	In patients with ARI and tested for virus	≥ 50; M, 66	508		6.1%	
**RSV Testing During Full Winter Season**									
**Hospitalized**									
Branche et al. [39]	PCR	Rochester, NY; Prospective cohort; Single-center	Nov 1 to May 30 in 2008–2012	Hospitalized with acute RTI	> 21; A, 63	965		6.5%	

A = average; ARI = acute respiratory illness; BAL = bronchoalveolar lavage fluid; CAP = community-acquired pneumonia; CRX = chest-x-ray–confirmed; DFA = direct immunofluorescence; ED = emergency department; ICU = intensive care unit; M = median; PCR = polymerase chain reaction; RSV = respiratory syncytial virus; RTI = respiratory tract infection. Shading in the Dates column indicates all-year testing for RSV (blue), testing for RSV in the whole winter season (tan), and testing for RSV during the influenza season (purple). Medically attended RSV is RSV-positive respiratory illness in which the patient was seen in a physician’s office, an emergency department, or a hospital.

^a^ Prospective implied, not stated overtly.

Full-year observation periods may diminish the estimated medically attended–RSV incidence proportions because the denominator has more cases of ARI from seasons when RSV was not prevalent than studies limited to the RSV season or influenza season. However, the six studies assessing RSV over the full year differed in the population studied, which likely impacted the incidence proportion estimated [23, 25–27, 31, 40]. The older adults studied in Sumino et al. [25] had one of the lowest estimated incidence proportions, including for those hospitalized with ARI for which bronchoscopy with microbiologic testing was considered necessary, which could exclude patients with RSV infection. Glezen et al. [31], with the lowest incidence proportion, was the oldest study and used only serology and viral culture to identify RSV infection. The older adults studied in Walker and Ison [26] were hospitalized and tested positive for a respiratory virus (excluding those with negative results and patients with bacterial infection), and had higher incidence proportions than the other studies. In the one study in an ICU population admitted for CAP that presented results by type A and B RSV, the incidence proportions were equal for types A and B RSV [27].

The studies during the influenza season were all in different clinical settings and during different calendar time periods, which would impact the incidence of influenza (Table 1) [24, 28, 29], and the outpatient-only study was limited to patients with upper respiratory tract infection [28].

Only one study assessed medically attended–RSV incidence proportion with a winter testing period [39]. This study included older adults hospitalized with ARI defined not only as new respiratory symptoms but also exacerbation of asthma or COPD for patients with these conditions (proportions not reported). The medically attended–RSV incidence proportion was 6.5%, similar to the 6.1% of hospitalized patients assessed during the influenza season in Widmer et al. [24].

### Incidence proportions of medically attended RSV in special populations of older adults

The proportion of patients with medically attended RSV has been investigated in a number of special populations (long-term care facility, chronic cardiopulmonary disorders, COPD, lung transplant). These special populations represent substantial numbers of persons.

Table 2 shows incidence proportions of medically attended RSV (and the overall incidence of RSV in community cohorts) estimated in nine studies in older adult populations selected for not having underlying health conditions (healthy elderly) [7], for having disability (i.e., long-term care facility residents) [30], and for having underlying health conditions [7, 12, 32–36, 38]. Like Table 1 for unselected older adults, Table 2 shows the heterogeneity of design in these studies and, thus, the difficulty in trying to compare medically attended–RSV incidence proportions among these studies.

**Table 2:** RSV incidence proportions by observational period and clinical setting in special populations of older adults.

Reference	RSV Test	Study Location, Design	Dates/ Observational Period	Clinical Setting	Population	Age in Years, Median (M) or Average (A)	N	RSV Incidence Proportions (Percentage of Patients)
**Community or Hospital Setting Cohorts**								
Falsey et al. [7]	PCR/‌culture/‌serology/ RSV IgG titers before and after season^a^	Rochester, NY; Prospective 3-cohort; Multicenter	1999–2003 winters (Nov 15-Apr 15)	Community	Healthy elderly (aged ≥ 65 years and no disabling underlying condition)	≥ 65; A, 75	212 280 180 295	1999–2000 6% any RSV 2000–2001 7% any RSV 2001–2002 3% any RSV 2002–2003 3% any RSV 17% of RSV had office visits 0% of RSV visited ED 0% of RSV hospitalized
				Community	High-risk adults (aged ≥ 21 years and chronic pulmonary disease or CHF)	≥ 21; A, 70	206 271 195 210	1999–2000 10% any RSV 2000–2001 7% any RSV 2001–2002 4% any RSV 2002–2003 5% any RSV 29% of RSV had office visits 9% of RSV visited ED 16% of RSV hospitalized
				Hospitalized	Aged ≥ 65 years; or > 21 years with underlying cardiopulmonary (85%) disease hospitalized with acute respiratory infection	≥ 65; A, 75	274 296 434 384	1999–2000 8% RSV 2000–2001 13% RSV 2001–2002 10% RSV 2002–2003 10% RSV
**Long-term Care Facility Residentss**								
Falsey et al. [30]	RSV IgG at baseline and 53 weeks	Boston, MA; Prospective; Multicenter	1998–2000	LTCF	LTCF residents aged ≥ 65 years not room bound or on long-term steroids	A, 84.9	617	RSV-positive cases at 53 weeks: 6.5%
					Virus-infected subset	NR	157	RSV-positive cases at 53 weeks: 15.9%
**Older Adults With Chronic Underlying Conditions from a Vaccine Clinical Trial**								
Falsey et al. [12]	PCR/ serology	38 centers throughout US; RCT; Multicenter	2 years, 2002–2003 and 2003–2004	Clinical trial of vaccines testing all with respiratory symptoms	Aged ≥ 65 years with cardiopulmonary disease (CHF, COPD, chronic bronchitis, emphysema, asthma, interstitial fibrosis, or environmental lung disease)	≥ 65; A, 74	Year 1: 1,169	Any symptomatic RSV, 3.1% Medically attended, 2.1% Hospitalized, 0.3%
							Year 2: 527	Any symptomatic RSV, 2.1% Medically attended, 1.1% Hospitalized, 0.0%
**Older Adults With COPD**								
Mehta et al. [33]	PCR/‌serology/‌viral culture	Rochester, NY; Retrospective (post hoc); Multicenter	Two of four winters in 1999–2003	2-cohort,^b^ longitudinal study (community, outpatient, ED, or hospital)	Monitored for respiratory illness (not medically attended RSV) or	A, 69.9	379	Any, 11.1% Medically attended, 5.3%
			and 12 months from enrollment in July-Oct 2004		Physician’s office, ED, or hospital with respiratory illness (medically attended RSV)			
Camargo et al. [38]	PCR	Minneapolis, MN, and Boston, MA; Prospective; Multicenter	Late Dec 2003-end of April 2004 (1 winter)	ED	Moderate-to-severe AECOPD	A, 72.0	76	7.9%
Beckham et al. [32]	PCR	Houston, TX, and Harris Country, TX; Retrospective (2 cohorts); Multicenter	July 1991-June 1995 (cohort 1); Sep 1991-May 1995 (cohort 2)	Outpatients or hospitalized	Cohort 1: AECOPD; Cohort 2: AECOPD	A, 63.1	96	3.6% (of 194 events)
Martinello et al. [34]	DFA	New Haven, CT; Prospective cohort; Single-center	Dec 13, 2002-May 6, 2003	Hospitalized	AECOPD	M, 70.0	50	8.0% (95% CI, 2%-19%)
**Lung Transplant Recipients (Immunocompromised)**								
Weinberg et al. [35]	PCR/‌viral culture	Denver, CO; Prospective cohort; Single center	Sep 2005-Nov 2007	NR (followed)	Lung transplant recipients with signs or symptoms^c^ of RTIs [7, 43]	M, 60.0	60; (112 RTIs)	11.6% (of RTIs)
Milstone et al. [36]	PCR/‌serology/‌viral culture	Nashville, TN; Prospective cohort; Single center	Nov 1, 1999-Mar 31, 2000	Outpatients	Lung transplant recipients	A, 50.0	50	16.0%

A = average; AECOPD = acute exacerbation of chronic obstructive pulmonary disease; ARI = acute respiratory infection; CHF = congestive heart failure; CI = confidence interval; COPD = chronic obstructive pulmonary disease; DFA = direct immunofluorescence; ED = emergency department; LTCF = long-term care facility; M = median; NR = not reported; PCR = polymerase chain reaction; RCT = randomized controlled trial; RSV = respiratory syncytial virus; RTI = respiratory tract infection; US = United States. Shading in the Dates/Observational Period column indicates all-year testing for RSV (blue), testing for RSV in the whole winter season (tan), and testing for RSV during the metapneumovirus winter season (purple). Medically attended RSV is RSV-positive respiratory illness in which the patient was seen in a physician’s office, an emergency department, or a hospital.

^a^ Signs and symptoms were those suggestive of upper respiratory tract infection (e.g., rhinorrhea, sore throat, or cough) or of lower respiratory tract infection (e.g., wheezing, a > 10% decline in forced expiratory volume in 1 second [FEV_1_]), shortness of breath, or oxygen).

^b^ RSV was detected in the entire study (N = 2,514) by PCR (93%), viral culture (93%), and serology (81%).

^c^ Patients with COPD from Falsey et al. [7] and all of the patients with COPD in Falsey et al. [43] were included in this analysis.

#### Healthy or high-risk elderly living in the community or hospitalized with an acute respiratory infection

In an observational study by Falsey et al. [7] spanning four consecutive winters (1999–2003), incidence proportions were estimated for three different population cohorts: adults aged ≥ 65 years who had no disabling underlying health conditions living in the community (healthy elderly), adults aged 21 years or more with cardiopulmonary conditions living in the community, and adults aged 65 or more years or aged 21 or more years with underlying cardiopulmonary conditions who were hospitalized with acute respiratory symptoms. During each season in the two community cohorts, enrollees were tested for RSV if they reported respiratory symptoms. RSV was also detected by testing immunoglobulin levels at the beginning and end of each season, which allowed detection of asymptomatic as well as symptomatic cases.

In each winter, 3% to 7% of the healthy elderly cohort in the Falsey et al. [7] study tested positive for RSV, with 89% being symptomatic across the four winters. None of the patients with RSV cases in this healthy elderly cohort required hospitalization, but 17% of cases across the four winters sought medical attention for RSV illness via a physician’s office visit, and 15% called the doctor [7]. In contrast to the healthy elderly cohort, 4% to 10% of the high-risk cohort living in the community tested positive for RSV across four winters, with 89% being symptomatic. In this cohort, as well as the somewhat higher incidence of RSV, its impact on need for medical care was greater, with 29% having office visits, 23% calling the doctor, 9% visiting the emergency department, and 16% requiring hospitalization. In the third, hospitalized, cohort, of whom 85% had high risk-conditions, the denominator was those hospitalized with acute respiratory symptoms, and the incidence proportions for RSV were a little higher than the high-risk community cohort, ranging from 8% to 13% over the 4-year study.

#### Long-term care facility residents

An estimated 1.4 million persons lived in nursing homes in the US in 2012 [44]. In 2010, approximately 1% of persons aged 65 to 74 years were institutionalized, and 13% of persons aged ≥ 85 years were institutionalized [44], or 935,000 adults aged 65–74 years plus adults aged ≥ 85 years (based on 2013 population data) [45]. In a 3-year, prospective surveillance cohort of long-term care facility residents aged ≥ 65 years in the US, 6.5% of the 617 residents were RSV positive by immunoglobulin levels determined at study entry and at 53 weeks (annual rate) [30]. Of those who were identified as having a viral infection during the year, 15.9% were positive for RSV infection.

#### Adults with chronic cardiopulmonary disorders from a vaccine clinical trial

In the US, the 5-year prevalence (2007–2011) of common chronic cardiopulmonary diseases among adults aged ≥ 65 years was 19% for coronary heart disease, nearly 10% each for stroke and COPD, and nearly 8% each for heart failure and asthma [46]. To give context to those proportions, in 2013, over 43 million persons in the US were aged ≥ 65 years [45].

In Falsey et al. [12], adults aged ≥ 65 years (mean age, 74 years) were enrolled in a clinical trial of RSV vaccines during the years 2002–2004. All enrollees with respiratory symptoms were tested for RSV infection. Any symptomatic RSV occurred in 3.1% in the year 1 cohort (2002–2003) and 2.1% in the year 2 (2003–2004) cohort. Medically attended RSV occurred in 2.1% of the year 1 clinical trial cohort and 1.1% of the year 2 clinical trial cohort. Of the year 1 cohort, 0.3% of patients were hospitalized due to RSV, whereas no patients in the year 2 cohort were hospitalized because of RSV [12]. Of note, in the high-risk community cohort from Falsey et al. [7], the symptomatic RSV rates were approximately 9% and 6% in the winters of 1999–2000 and 2000–2001, falling to 4% each winter in 2001–2002 and 2002–2003, assuming that 89% were symptomatic in each year; thus, estimates of a symptomatic RSV illness rates in the chronic illness groups in the same geographic area in 2002 to 2004 may have been at a low point during the vaccine trial.

#### COPD

In 2008, the discharge rates per 100,000 population for acute exacerbation of chronic obstructive pulmonary disease (AECOPD) were 770 for persons aged 65 to 74 years, 1,075 for persons aged 75 to 84 years, and 913 for persons aged ≥ 85 years [47]. Using these hospitalization rates, over 380,000 persons aged ≥ 65 years were hospitalized with AECOPD (based on 2013 population data [45]).

Of the four studies of RSV incidence in older adults with AECOPD, Beckham et al. [32] assessed patients throughout the study year; Camargo et al. [38] assessed patients throughout the winter; Martinello et al. [34] evaluated patients during a metapneumovirus season (in winter); and Mehta et al. [33] combined patients from two cohorts, one cohort followed in the winter [7] and one with RSV testing throughout the year [43].

None of the studies of COPD required fever as a symptom among patients tested for RSV. Also, none of the studies were limited to patients with symptoms of infection (e.g., sputum purulence), instead also including patients with exacerbations whose usual level of dyspnea, sputum production, and/or cough were increased [32–34, 38].

Three meta-analyses based on systematic reviews not limited by country have reported RSV incidence proportions of 5.3% [48], 9.0% [49], and 9.0% [50] among patients with COPD, which is similar to that reported in this review except for Beckham et al. [32], which assessed the incidence proportion over a complete year versus just the winter, which would tend to lower the rate of RSV. In multivariate analysis in the study by Mehta et al. [33], congestive heart failure was the only factor significantly associated with medically attended RSV (odds ratio, 4.16; 95% CI, 1.02–17.01).

The role of RSV in COPD may be more complicated than simply triggering acute exacerbations with new infections. Studies of patients with stable COPD suggest that RSV may represent a persistent infection, with low viral loads [51, 52], that may contribute to chronic inflammation, contributing to greater severity of COPD [51]. When patients with stable COPD were stratified by frequency of testing positive for RSV, those testing positive more than half of the time had higher levels of airway inflammation and faster levels of decline in forced expiratory volume in the first second [53].

#### Lung transplant recipients

Two studies determined RSV incidence proportions in lung transplant recipients: one with all-year testing over 2 years [35] and one testing only in one winter [36]. Among 60 lung transplant recipients with a median age of 60 years and prospectively followed for upper and/or lower respiratory tract infection, there were 112 respiratory tract infections, and 11.6% of these were due to RSV [35]. In the other study, 50 lung transplant recipients (median age, 50 years) were followed in an outpatient clinic for one winter [36]. Of these, 32 developed signs or symptoms of upper and/or lower respiratory tract infection, and 8 tested positive for RSV (25% of the ill patients and 16% of the cohort). Based on these small studies, it appears that RSV represents a considerable concern for lung transplant recipients, many of whom are older in addition to having undergone lung transplant.

#### Immunocompromised patients and patients With asthma

This systematic review did not identify studies that reported a medically attended–RSV incidence proportion or rate among older adults with asthma or who were immunocompromised from causes other than lung transplant.

### Resource use in hospitalized older adults with RSV

Length of hospitalization for mostly older populations with RSV generally ranged from 3 to 6 days [23, 24, 37] but was 14 days in a hospitalized cohort of mean age 76 years [7] (Table 3). However, the mean stay of 14 days was skewed because 1 of the 132 patients hospitalized with RSV stayed in the hospital 450 days. [54] In five studies, 10% to 31% of RSV-positive hospitalized older adults were admitted to the intensive care unit [7, 23, 24, 26, 37] (Table 3). Use of mechanical ventilation was required for 4% of older adults with RSV seen in the ED or hospital [23] and in 3% to 17% of patients hospitalized with ARI who tested positive for RSV [7, 23, 24] (Table 3).

**Table 3:** Resource use by older adults hospitalized with RSV.

Reference	Study Period	Patient Characteristics	N	Age (Years)	Hospitalization Length of Stay by RSV-Positive Patients (Days)	ICU Admission (%)	Mechanical Ventilation (%)
Falsey et al. [7]	4 consecutive winters (1999–2003)	Hospitalized with acute cardiopulmonary illness and RSV positive	132	Average, 76	Mean (SD), 14 (41)^a^	15	13
Lee et al. [37]	3 consecutive winters (2005–2008)	Hospitalized with RSV ■ Steroid treated	33	Average, 69.8	Mean (SD), 6.2 (5.6)	18	NR
		Hospitalized with RSV ■ Not steroid treated	17	Average, 72.0	Mean (SD), 5.9 (3.2)	29	NR
Walker and Ison [26]	April 2009 to March 2010	Hospitalized adults with a positive molecular respiratory virus assay	48	≥ 18 years; 65% of patients with RSV were aged ≥ 60	NR	31	17.0
Widmer et al. [24]	3 influenza seasons (2006–2009)	Hospitalized with ARI and tested for virus	31	Median, 68	Median (IQR), 3 (2–6)	10	3
Widmer et al. [23]	May 2009 to April 2010	Patients in the emergency department or hospitalized for respiratory symptoms	24	NR^b^	Median (25%, 75%), 4 (2, 5)	17	4

ARI = acute respiratory infection; ICU = intensive care unit; IQR = interquartile range; NR = not reported; RSV = respiratory syncytial virus; SD = standard deviation.

^a^ One patient was hospitalized for 450 days.

^b ^The median age of the 32 patients in this study was 60.8 years. Of the 32 patients, 24 were hospitalized [23].

### Mortality due to RSV in unselected populations of older adults

**Fig 3:**
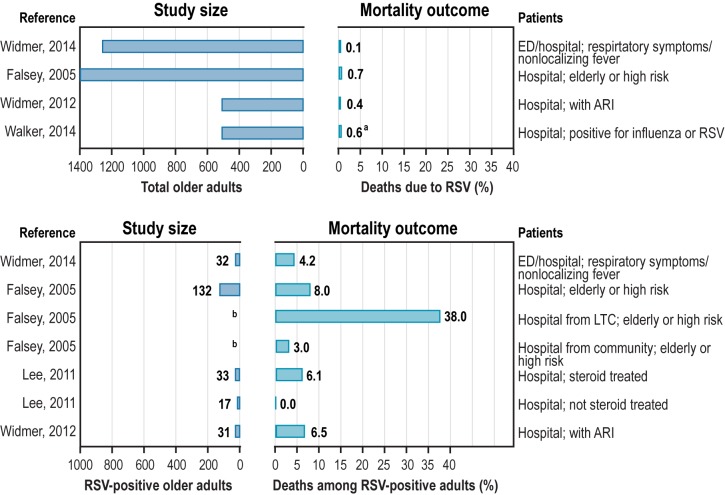
Mortality due to RSV in older adults from special populations, by population and clinical setting. ARI = acute respiratory illness; ED = emergency department; LTC = long-term care; RSV = respiratory syncytial virus. Note: The case fatality ratio in the bottom right panel of the figure shows the proportion of RSV-positive older adults who died. ^a^ Two deaths and one patient discharged to hospice. ^b^ Sample size not reported.

None of the studies using laboratory confirmation of RSV that estimated mortality rates were national studies or studies in special populations. ‎Fig 3 shows the mortality due to RSV in older adults by clinical setting; the left panels display the size of the studies.

The top part of Fig 3 shows the proportion of patients who died from RSV among larger cohorts of patients with any respiratory illnesses (> 500). Among these larger cohorts, the proportion of deaths associated with RSV was 0.1% to 0.7% [7, 23, 24, 26]. In Widmer et al. [23], there was one RSV death among 1,248 older adults admitted to the ED or hospital with acute respiratory symptoms or nonlocalizing fever. Among the 1,388 elderly or high-risk patients hospitalized in Falsey et al. [7] plus the 9 hospitalized patients from the high-risk community cohort in that study, 10 patients died with RSV infection (0.7%). Among 508 older adults hospitalized with ARI and tested for virus in Widmer et al. [24], 2 died due to RSV (0.4%). In Walker and Ison [26], among 502 hospitalized patients testing positive on influenza or a respiratory virus panel assay, 2 died with RSV infection (0.4%) and one was discharged to hospice (total deaths or anticipated deaths, 0.6%).

The lower section of ‎Fig 3 shows the proportion of RSV-positive patients who died in the ED or hospital setting (the case fatality ratio or proportion). In Widmer et al. [23], 1 of 32 (4.2%) RSV-positive patients died. Of these 32 patients, 88% had at least one chronic illness, 69% had chronic pulmonary disease, 59% had immunodeficiency, and 47% had cardiovascular disease. In a US study by Falsey et al. [7], 8% of 132 elderly or high-risk RSV-positive adults died. In this study, the proportion of patients who died from RSV was much higher (38%) for those admitted to the hospital from a long-term care facility compared with those admitted from the community (3%) [7]. In Widmer et al. [24], 2 of 31 (6.5%) RSV-positive patients died. Of the 31 patients, most had at least one chronic condition. Among older adults admitted to the hospital with upper or lower respiratory tract diseases, Lee et al. [37] found that none of the 17 RSV-positive patients not treated with steroids died, whereas 2 (6.1%) of the 33 RSV-positive patients treated with steroids died.

### Limitations of the review

This review was intended to be qualitative, and no statistical synthesis of the data was planned. In addition, given the large variability in case definitions for acute respiratory illness, clinical settings, observational periods for this seasonal disease, geographic regions, and age group breakouts of the data, we would expect to have large heterogeneity when combining the data, making a meta-analysis not feasible.

In addition to the differences in study parameters, incidence of the disease in adults is known to vary annually. Taken together, these differences may explain the considerable variation in RSV incidence estimates in the studies reported in this review among studies reporting a similar outcome (e.g., incidence per 10,000 age-specific population).

Most of the identified studies tested for RSV in populations from limited locales, primarily in the eastern US, and only one study claimed to be nationally representative [17]. Actual surveillance of RSV in older adults with serologic and/or PCR testing would provide more accurate estimates of the disease burden in this population.

Our literature review did not identify any studies that reported RSV incidence by the number of comorbidities patients had, although overall proportions with individual categories of comorbidities were reported in many. Most studies in this review included specific testing for viral etiology, and one virus for which they tested was RSV. The tables of patient demographics in the reports listed the proportion of patients with individual comorbidities, but only a few studies reported the comorbidities specifically in patients with RSV [7, 23, 24, 37]. In addition, we did not report any findings on the impact on disease severity of coinfection with multiple viruses. A patient with two concomitant respiratory infections may have clinical illness from both microbial agents, or just one of them. Studies in this review varied in their reporting of coinfection, with most studies that reported multiple infections giving only a proportion, which was a minority of RSV cases, with no discussion of timing or impact on illness. The exception was Walker and Ison [26], which noted that most patients with respiratory viral infections, including RSV, who required ventilator support had a documented or suspected bacterial or fungal coinfection.

This literature review also did not identify any US-based studies of long-term outcomes of RSV infection in older adults. One Chinese study has reported a significant association between RSV infection and risk of acute myocardial infarction in elderly patients, although the exact mechanism was unclear [55].

## Conclusions

Medically attended–RSV infection was detected in up to 12% of older adults with an ARI and not selected for underlying medical conditions. Both the observational and clinical trial studies by Falsey and colleagues [7, 12, 30] demonstrate the variability in RSV infection rates in different calendar years in several different population cohorts. Medically attended–RSV infection appears to become increasingly common with advancing age, and is associated with substantial morbidity and health care resource use. Although mortality is limited for those receiving only outpatient care (< 1%), the risk of mortality is markedly increased among older adults who are hospitalized with RSV-associated illnesses: approximately 6% to 8%, on average, for the studies in this review. Most patients hospitalized with ARI who tested positive for RSV in these studies had pulmonary, cardiac, and/or immunodeficiency conditions that would increase their risk of complications following any respiratory tract infection.

Protection of older adults against RSV could reduce respiratory-related burden, especially for more elderly patients and patients with comorbidities. Increased awareness of RSV as an important pathogen of older adults should lead to better definition of its overall burden through use of the highly sensitive molecular diagnostics now available and should serve to incentivize development of vaccines and/or therapeutics.

## Supporting information

S1 Table Search strategy: PubMed. (PDF)

S2 Table Critical appraisal of cohort studies reporting RSV epidemiology and health s1 care resource in older adults. (PDF)

S3 Table Critical appraisal of a randomized controlled trial reporting RSV epidemiology in older adults with chronic cardiopulmonary disorders. (PDF)

S4 Table Critical appraisal of a randomized controlled trial reporting health care utilization related to RSV in older adults with chronic cardiopulmonary disorders. (PDF)

S1 File PRISMA checklist. (DOC)
