# Supplementary material for: The epidemiology of medically attended respiratory syncytial virus in older adults in the United States: A systematic review
Source: PLoS One. 2017 Aug 10;12(8):e0182321. doi: 10.1371/journal.pone.0182321 (PMC5552193; doi:10.1371/journal.pone.0182321)
Supplement: S1 Table — (PDF) [file pone.0182321.s001.pdf]

## S1 Table. Search Strategy: PubMed

| Search No. | Search Terms | No. of Results |
|---|---|---|
| **Disease Terms** | | |
| 1 | "Respiratory Syncytial Virus, Human"[Mesh] OR "Respiratory Syncytial Virus Infections"[Mesh] OR "Respiratory Syncytial Virus"[Title] OR "RSV"[Title] OR ("Respiratory Tract Infections"[Mesh] AND (syncytial[Text Word] OR RSV [Text Word])) Limits: Publication date up to 03/11/2016 | 10,811 |
| **Epidemiology Terms** | | |
| 2 | #1 AND ("Respiratory Syncytial Virus Infections/epidemiology"[Mesh] OR "Epidemiology"[Mesh] OR "Pharmacoepidemiology"[Mesh] OR "Morbidity"[Mesh] OR "Incidence"[Mesh] OR "Prevalence"[Mesh] OR "Disease Progression"[Mesh] OR "Mortality"[Mesh] OR ("Frail Elderly"[Mesh] or frail*[tiab]) OR epidemiolog*[Title] OR pharmacoepidemiolog*[Title] OR morbidity[Title] OR incidence[Title] OR prevalence[Title] OR "disease progression"[Title] OR mortality[Title]) Limits: Publication date up to 03/11/2016 | 2,139 |
| **Symptoms** | | |
| 3 | #1 AND ("Signs and Symptoms"[Majr] OR symptom*[Title]) Limits: Publication date up to 03/11/2016 | 327 |
| **Economic Terms** | | |
| 4 | #1 AND ("Respiratory Syncytial Virus Infections/economics"[Majr] OR "Costs and Cost Analysis"[Majr] OR "Cost-Benefit Analysis"[Majr] OR "Economics"[Majr] OR "Economics, Hospital"[Majr] OR "Economics, Medical"[Majr] OR "Economics, Nursing"[Majr] OR "Economics, Pharmaceutical"[Majr] OR "Health Resources/utilization"[Majr] OR "Fees and Charges"[Majr] OR "Models, Economic"[Majr] OR "Employment"[Majr] OR "Work"[Majr] OR "Health Care Costs"[Majr] OR "Cost of Illness"[Majr] OR ((economic*[Title]) AND burden*[Title]) OR hospitalization*[Title] OR hospitalisation*[Title] OR economic*[Title] OR price*[Title] OR pricing[Title] OR cost[Title] OR costs[Title] OR "cost analysis"[Title] OR cost effective*[Title] OR "cost utility"[Title] OR "economic model"[Title] OR pharmacoeconomic*[Title] OR modeling[Title] OR modelling[Title] OR "economic model"[Title] OR "resource use"[Title] OR "resource utilization"[Title] OR "resource utilisation"[Title] OR health care cost*[Title] OR productivity[Title] OR "cost-minimization"[Title] OR "cost-minimisation"[Title] OR "cost-minimisation analysis"[Title] OR productivity cost*[Title] OR societal cost*[Title] OR economic benefit*[Title] OR "employment"[Title] OR "unemployment"[Title] OR (cost*[Title] AND (effective*[Title] OR utilit*[Title] OR benefit*[Title] OR minimi*[Title])) OR "economic evaluation"[Title] OR "cost effectiveness analysis"[Title]) Limits: Publication date up to 03/11/2016 | 427 |
| **Treatment** | | |
| 5 | #1 AND ("Respiratory Syncytial Virus Infections/drug therapy"[Majr] OR "Therapeutics"[Majr] OR "Practice Patterns, Physicians'"[Majr] OR "Practice Guidelines as Topic"[Majr] OR "Drug Utilization"[Majr] OR treat*[Title] OR therap*[Title] OR practice pattern*[Title] OR guideline*[Title] OR "Practice Guideline" [Publication Type]) Limits: Publication date up to 03/11/2016 | 1,494 |
| **Health-Related Quality of Life** | | |
| 6 | #1 AND ("Quality of Life"[Mesh] OR "Patient Satisfaction"[Mesh] OR "Pain Measurement"[Mesh] OR "Questionnaires"[Mesh] OR "Activities of Daily Living"[Mesh] OR "Treatment Outcome"[Mesh] OR "Patient Compliance"[Mesh] OR "Medication Adherence"[Mesh] OR "Caregivers"[Mesh] OR "quality of life"[Title] OR "QoL"[Title] OR "hrqol"[Title] OR "hqol"[Title] OR "hrql"[Title] OR patient report*[Title] OR self report*[Title] OR "health utility"[Title] OR "health utilities"[Title] OR "standard gamble"[Title] OR "time trade off"[Title] OR "TTO"[Title] OR "EuroQol"[Title] OR "EQ5D"[Title] OR "EQ 5D"[Title] OR "quality of well being"[Title] OR "HUI"[Title] OR "SF-6D"[Title] OR "QALY"[Title] OR "quality adjusted life year"[Title] OR "quality adjusted life years"[Title] OR "SF-36"[Title] OR "sf36"[Title] OR "activities of daily living"[Title] OR "patient satisfaction"[Title] OR | 500 |

| Search No. | Search Terms | No. of Results |
|---|---|---|
| | burden[Title] OR "sf-12"[Title] OR "sf12"[Title] OR "health status"[Title] OR questionnaire*[Title] OR survey*[Title] OR "functional status"[Title] OR physical function*[Title] OR carer*[Title] OR caregiver*[Title] OR care giver*[Title] OR "medical burden"[Title]) Limits: Publication date up to 03/11/2016 | |
| **Limits** | | |
| 7 | "Animals"[Mesh] NOT "Humans"[Mesh] Limits: Publication date up to 03/11/2016 | 4,212,344 |
| 8 | mice[title] NOT humans[title] Limits: Publication date up to 03/11/2016 | 245,458 |
| 9 | "Clinical Trials, Phase I as Topic"[Mesh] OR "Editorial" [Publication Type] OR "Comment" [Publication Type] OR "Legislation" [Publication Type] OR "Letter"[Publication Type] OR "Clinical Trial, Phase I"[Publication Type] Limits: Publication date up to 03/11/2016 | 1,492,404 |
| 10 | ("Child"[Mesh] OR "Infant"[Mesh] OR "Adolescent"[Mesh] OR "Young Adult"[Mesh] OR infant*[Title] OR child*[Title] OR adolescen*[Title] OR teen*[Title] OR youth*[Title] OR "school age"[Title]) NOT ("Aged"[Mesh] OR "Aged, 80 and over"[Mesh] OR "Frail Elderly"[Mesh] OR "Middle Aged"[Mesh] OR "old age"[Title] OR senior citizen*[Title] OR elderly[Title] OR older adult*[Title]) Limits: Publication date up to 03/11/2016 | 2,406,579 |
| 11 | "in vitro"[Title] OR cell line*[Title] Limits: Publication date up to 03/11/2016 | 324,717 |
| 12 | "Case Reports" [Publication Type] OR "case study"[Text Word] OR "case studies"[Text Word] Limits: Publication date up to 03/11/2016 | 1,833,550 |
| **Combined** | | |
| 13 | (#2 OR #3 OR #4 OR #5 OR #6) | 3,803 |
| 14 | #13 NOT (#7 OR #8 OR #9 OR #10 OR #11 OR #12) | 859 |
| 15 | #14 AND Publication date from 2000/01/01 | 681 |
| 16 | #15 NOT "Review"[Publication Type] | 507 |
| 17 | #15 AND "Review"[Publication Type] AND Publication date from 2010/01/01 | 63 |
| 18 | #16 OR #17 | **570** |

**Limits:** Aged or middle-aged; no letters, editorials, comments, case reports, legislation, or phase 1 clinical trials; time limits of 2000 forward (past 15 years) for nonreview articles and 2010 forward (past 5 years) for review articles.
