# Supplementary material for: The epidemiology of medically attended respiratory syncytial virus in older adults in the United States: A systematic review
Source: PLoS One. 2017 Aug 10;12(8):e0182321. doi: 10.1371/journal.pone.0182321 (PMC5552193; doi:10.1371/journal.pone.0182321)
Supplement: S2 Table — (PDF) [file pone.0182321.s002.pdf]

Critical Appraisal

Because the case definition of respiratory syncytial virus (RSV) was critical to this review, studies were organized by the sensitivity of the laboratory techniques for RSV detection, based on a sensitivity ranking from Talbot and Falsey [1]. For this systematic literature review, we ranked studies that tested all patients by polymerase chain reaction (PCR)–based methods and/or serology as best defining the RSV case definition, followed by studies that used a mix of laboratory methods that included PCR and/or serology but not for all patients, and studies that did not test any patients with PCR or serology. Other studies that used statistical modeling of the winter virus seasons and diagnosis, discharge, or death certificate codes were excluded from this review. The size of the older adult population within studies was also noted (large, > 300; medium, 101-300; small, 50-100; and very small < 50).

S2 Table. Critical Appraisal of Cohort Studies Reporting RSV Epidemiology and Health Care Resource in Older Adults

Reference	Reliable RSV Test	Question Focused	Tells How Many Asked vs. Did[a]	Clear Outcomes	Separate Older Age Group	CIs	Prospective or Retrospective	SC vs. MC	Nationally Representative	Sample Size
**RSV Incidence / Prevalence in Older Adults Unselected for Chronic Health Conditions**										
**All-Year RSV Testing**										
**Outpatient Setting**										
Fowlkes et al. [2]	Yes	Yes	Yes	Yes	Yes	Yes	P[b]	MC	Yes	Large
**Mix of Clinical Settings (Outpatient and Inpatient)**										
Widmer et al. [3]	Yes	Yes	Yes	Yes	Yes	Yes	P	MC	No	Large
**Hospitalized Setting**										
Sumino et al. [4]	Yes	Yes	Yes	Yes	No	No	P[b]	SC	No	Medium
Walker and Ison [5]	Yes	Yes	NA[c]	Yes	Yes	No	R	MC	No	Medium
Jain et al. [6]	Yes	Yes	Yes	Yes	Yes	Yes	P	MC	No	Large
**Intensive Care Unit Setting**										
Wiemken et al. [7]	Yes	Yes	NA[b]	Yes	No	No	P	MC	No	Large
**RSV Testing in Influenza Season**										
**Outpatient Setting**										
Zimmerman et al. [8]	Yes	Yes	No	Yes	Yes	No	P	SC	No	Medium
**Mix of Clinical Settings (Outpatient and Inpatient)**										
McClure et al. [9]	Yes	Yes	No	Yes	Yes	Yes	P	MC	No	Large
Sundaram et al. [10]	Yes	Yes	Yes	Yes	No	No	P	MC	No	Large
**Hospitalized Setting**										
Widmer et al. [11]	Yes	Yes	Yes	Yes	Yes	Yes	P	MC	No	Large
**RSV Testing All Winter**										
**Hospitalized Setting**										
Branche et al. [12]	Yes	Yes	No	Yes	Yes	No	P	SC	No	Large

Reference	Reliable RSV Test	Question Focused	Tells How Many Asked vs. Did[a]	Clear Outcomes	Separate Older Age Group	CIs	Prospective or Retrospective	SC vs. MC	Nationally Representative	Sample Size
**RSV Incidence / Prevalence in Older Adults Selected for Absence or Presence of Chronic Health Conditions**										
**Healthy Elderly**										
**Mix of Clinical Settings (Outpatient and Inpatient)**										
Falsey et al. [13]	Yes	Yes	Yes	Yes	Yes	No	P	MC	No	Large
**Chronic Cardiopulmonary Disorders**										
**Mix of Clinical Settings (Outpatient and Inpatient)**										
Falsey et al. [13]	Yes	Yes	Yes	Yes	Yes	No	P	MC	No	Large
Glezen et al. [14]	Mixed	Yes	No	Yes	Yes	No	P	MC	No	Medium
**Nursing Home Residents**										
Falsey et al. [15]	Yes	Yes	Yes	Yes	Yes	No	P	MC	No	Large
Falsey et al. [13]	Yes	Yes	Yes	Yes	Yes and no[d]	Yes	P	MC	No	Large
**Patients With COPD**										
Beckham et al. [16]	Yes	Yes	No	Yes	No	No	R	MC[c]	No	Medium
Camargo et al. [17]	Yes	Yes	No	Yes	No	No	P	MC	No	Small
Mehta et al. [18]	Mixed	Yes	No	Yes	No	No	R[e]	Can't say	Can't say	Large
Martinello et al. [19]	Less so	Yes	Yes	Yes	No	Yes	P	SC	No	Small
**Patients With Asthma**										
Falsey et al. [13]	Yes	Yes	Yes	Yes	Yes	No	P	MC	No	Large
**Patients With Lung Transplants**										
Weinberg et al. [20]	Yes	Yes	No	Yes	No	No	P	SC	No	Small
Milstone et al. [21]	Mixed	Yes	No	Yes	No	No	P	SC	No	Small

Reference	Reliable RSV Test	Question Focused	Tells How Many Asked vs. Did[a]	Clear Outcomes	Separate Older Age Group	CIs	Prospective or Retrospective	SC vs. MC	Nationally Representative	Sample Size
**Mortality**										
**Nonnational Mortality, Inpatient, and Outpatient Settings**										
Widmer et al. [3]	Yes	Yes	Yes	Yes	No	No	P	MC	No	Large
Falsey et al. [13]	Yes	Yes	Yes	Yes	Yes	No	P	MC	No	Large
Lee et al. [22]	Yes	Yes	No	Yes	No	No	P	SC	No	Small
Widmer et al. [11]	Yes	Yes	Yes	Yes	No	No	P	MC	No	Large
Walker and Ison [5]	Yes	Yes	NA	Yes	Yes	No	R	MC	No	Medium
**Health Care Resource Use**										
Lee et al. [22]	Yes	Yes	Yes	Yes	Yes	No	Can't say	SC	No	Very small
Walker and Ison [5]	Yes	Yes	NA[f]	Yes	Yes	No	R	MC	No	Medium
Widmer et al. [3]	Yes	Yes	Yes	Yes	Yes	Yes	P	MC	No	Very small
Widmer et al. [11]	Yes	Yes	Yes	Yes	Yes	No	P	MC	No	Very small
Falsey et al. [13]	Yes	Yes	Yes	Yes	Yes	Yes	P	MC	No	Large

CIs = confidence intervals; COPD = chronic obstructive pulmonary disease; MC = multicenter; NA = not applicable; P = prospective; R = retrospective; RSV = respiratory syncytial virus; SC = single center.

[a] The study indicates how many of the people asked to take part did so, in each of the groups being studied.

[b] Implied, not stated overtly in Walker and Ison [5].

[c] Two cohorts.

[d] Patients were either older or high risk; the mean age of the high-risk cohort was 70 Falsey et al. [13].

[e] Two prospectively followed cohorts; COPD subset analyzed retrospectively.

[f] Retrospective.

References

1. Talbot HK, Falsey AR. The diagnosis of viral respiratory disease in older adults. Clin Infect Dis. 2010; 50 (5): 747-751.

2. Fowlkes A, Giorgi A, Erdman D, Temte J, Goodin K, Di Lonardo S, et al. Viruses associated with acute respiratory infections and influenza-like illness among outpatients from the influenza incidence surveillance project, 2010-2011. J Infect Dis. 2014; 209 (11): 1715-1725.

3. Widmer K, Griffin MR, Zhu Y, Williams JV, Talbot HK. Respiratory syncytial virus- and human metapneumovirus-associated emergency department and hospital burden in adults. Influenza Other Respir Viruses. 2014; 8 (3): 347-352.

4. Sumino KC, Walter MJ, Mikols CL, Thompson SA, Gaudreault-Keener M, Arens MQ, et al. Detection of respiratory viruses and the associated chemokine responses in serious acute respiratory illness. Thorax. 2010; 65 (7): 639-644.

5. Walker E, Ison MG. Respiratory viral infections among hospitalized adults: experience of a single tertiary healthcare hospital. Influenza Other Respir Viruses. 2014; 8 (3): 282-292.

6. Jain S, Self WH, Wunderink RG, Fakhran S, Balk R, Bramley AM, et al. Community-acquired pneumonia requiring hospitalization among U.S. adults. N Engl J Med. 2015; 373 (5): 415-427.

7. Wiemken T, Peyrani P, Bryant K, Kelley RR, Summersgill J, Arnold F, et al. Incidence of respiratory viruses in patients with community-acquired pneumonia admitted to the intensive care unit: results from the Severe Influenza Pneumonia Surveillance (SIPS) project. Eur J Clin Microbiol Infect Dis. 2013; 32 (5): 705-710.

8. Zimmerman RK, Rinaldo CR, Nowalk MP, Balasubramani GK, Thompson MG, Moehling KK, et al. Influenza and other respiratory virus infections in outpatients with medically attended acute respiratory infection during the 2011-12 influenza season. Influenza Other Respir Viruses. 2014; 8 (4): 397-405.

9. McClure DL, Kieke BA, Sundaram ME, Simpson MD, Meece JK, Sifakis F, et al. Seasonal incidence of medically attended respiratory syncytial virus infection in a community cohort of adults ≥50 years old. PLoS One. 2014; 9 (7): e102586.

10. Sundaram ME, Meece JK, Sifakis F, Gasser RA, Jr., Belongia EA. Medically attended respiratory syncytial virus infections in adults aged ≥ 50 years: clinical characteristics and outcomes. Clin Infect Dis. 2014; 58 (3): 342-349.

11. Widmer K, Zhu Y, Williams JV, Griffin MR, Edwards KM, Talbot HK. Rates of hospitalizations for respiratory syncytial virus, human metapneumovirus, and influenza virus in older adults. J Infect Dis. 2012; 206 (1): 56-62.

12. Branche AR, Walsh EE, Formica MA, Falsey AR. Detection of respiratory viruses in sputum from adults by use of automated multiplex PCR. J Clin Microbiol. 2014; 52 (10): 3590-3596.

13. Falsey AR, Hennessey PA, Formica MA, Cox C, Walsh EE. Respiratory syncytial virus infection in elderly and high-risk adults. N Engl J Med. 2005; 352 (17): 1749-1759.

14. Glezen WP, Greenberg SB, Atmar RL, Piedra PA, Couch RB. Impact of respiratory virus infections on persons with chronic underlying conditions. JAMA. 2000; 283 (4): 499-505.

15. Falsey AR, Dallal GE, Formica MA, Andolina GG, Hamer DH, Leka LL, et al. Long-term care facilities: a cornucopia of viral pathogens. J Am Geriatr Soc. 2008; 56 (7): 1281-1285.

16. Beckham JD, Cadena A, Lin J, Piedra PA, Glezen WP, Greenberg SB, et al. Respiratory viral infections in patients with chronic, obstructive pulmonary disease. J Infect. 2005; 50 (4): 322-330.

17. Camargo CA, Jr., Ginde AA, Clark S, Cartwright CP, Falsey AR, Niewoehner DE. Viral pathogens in acute exacerbations of chronic obstructive pulmonary disease. Intern Emerg Med. 2008; 3 (4): 355-359.

18. Mehta J, Walsh EE, Mahadevia PJ, Falsey AR. Risk factors for respiratory syncytial virus illness among patients with chronic obstructive pulmonary disease. COPD. 2013; 10 (3): 293-299.

19. Martinello RA, Esper F, Weibel C, Ferguson D, Landry ML, Kahn JS. Human metapneumovirus and exacerbations of chronic obstructive pulmonary disease. J Infect. 2006; 53 (4): 248-254.

20. Weinberg A, Lyu DM, Li S, Marquesen J, Zamora MR. Incidence and morbidity of human metapneumovirus and other community-acquired respiratory viruses in lung transplant recipients. Transpl Infect Dis. 2010; 12 (4): 330-335.

21. Milstone AP, Brumble LM, Barnes J, Estes W, Loyd JE, Pierson RN, 3rd, et al. A single-season prospective study of respiratory viral infections in lung transplant recipients. Eur Respir J. 2006; 28 (1): 131-137.

22. Lee FE, Walsh EE, Falsey AR. The effect of steroid use in hospitalized adults with respiratory syncytial virus-related illness. Chest. 2011; 140 (5): 1155-1161.
