# Supplementary material for: The epidemiology of medically attended respiratory syncytial virus in older adults in the United States: A systematic review
Source: PLoS One. 2017 Aug 10;12(8):e0182321. doi: 10.1371/journal.pone.0182321 (PMC5552193; doi:10.1371/journal.pone.0182321)
Supplement: S4 Table — (PDF) [file pone.0182321.s004.pdf]

S4 Table. Critical Appraisal of a Randomized Controlled Trial Reporting Health Care Utilization Related to RSV in Older Adults With Chronic Cardiopulmonary Disorders

Reference	RSV Test Reliability	Question Focused	Sample Size	Separate Older Age Group	Random Assignment	Adequate Concealment	Double Blind	Treatment Groups Similar at Start	Only Between-Group Difference is [illegible]	Standard, Reliable Outcome Measures	Percentage of Dropouts	ITT Analysis	Comparable Per-Site Results
Falsey et al. [1]	Yes	Yes	Medium	No	Yes	Can't say	Yes	Yes	Yes	Yes	Year 1, 17%<br>Year 2, 91%	Yes	Can't say

ITT = intention to treat; RSV = respiratory syncytial virus.

Reference

1. Falsey AR, Walsh EE, Capellan J, Gravenstein S, Zambon M, Yau E, et al. Comparison of the safety and immunogenicity of 2 respiratory syncytial virus (RSV) vaccines—nonadjuvanted vaccine or vaccine adjuvanted with alum—given concomitantly with influenza vaccine to high-risk elderly individuals. J Infect Dis. 2008; 198 (9): 1317-1326.
